# Long-term outcomes with intensive induction chemotherapy (carboplatin, bleomycin, vincristine and cisplatin/bleomycin, etoposide and cisplatin) and standard bleomycin, etoposide and cisplatin in poor prognosis germ cell tumours: A randomised phase II trial (ISRCTN53643604)

**DOI:** 10.1016/j.ejca.2019.12.028

**Published:** 2020-03

**Authors:** Fay H. Cafferty, Jeff D. White, Jonathan Shamash, Ivo Hennig, Sally P. Stenning, Robert A. Huddart

**Affiliations:** a MRC Clinical Trials Unit at UCL, 90 High Holborn, London, WC1V 6LJ, UK; b Beatson West of Scotland Cancer Centre, 1053 Great Western Road, Glasgow, G12 0YN, UK; c St Bartholomew's Hospital, West Smithfield, London, EC1A 7BE, UK; d Nottingham University Hospitals NHS Trust, Hucknall Road, Nottingham, NG5 1PB, UK; e The Institute of Cancer Research and the Royal Marsden NHS Foundation Trust, Downs Road, Sutton, SM2 5PT, UK

**Keywords:** Germ cell tumours, Poor prognosis, Randomised trial, Bleomycin

## Abstract

**Background:**

Up to 50% of men with poor prognosis, non-seminoma germ cell tumours (GCTs) die with standard BEP (bleomycin, etoposide and cisplatin) chemotherapy. An intensive regimen, CBOP/BEP (carboplatin, bleomycin, vincristine and cisplatin/BEP), met response targets in a randomised, phase II trial (74% complete response or partial response marker negative, 90% confidence interval (CI) 61%–85%).

**Aim:**

To assess long-term outcomes and late toxicity associated with CBOP/BEP.

**Methods:**

Patients with poor prognosis extracranial GCT were randomised to 4xBEP or CBOP/BEP (2xCBOP, 2xBO, 3xBEP with 15,000iu of bleomycin). Low-dose, stabilising chemotherapy before entry was permitted. Response rates (primary outcome) were reported previously. Here, we report secondary outcomes: progression-free survival (PFS), overall survival (OS) and late toxicity. Prognostic factors and the impact of marker decline are assessed in exploratory analysis.

**Results:**

Eighty-nine patients (43 CBOP/BEP) were randomised. After median 63 months follow-up, 3-year PFS is 55.7% (95% CI: 39.7%, 69.0%) for CBOP/BEP and 38.7% (95% CI: 24.7%, 52.4%) for BEP (hazard ratio [HR]: 0.59 (0.33, 1.06), p = 0.079). Three-year OS is 65.0% (48.8%, 77.2%) and 58.5% (43.0%, 71.2%), respectively (HR: 0.79 (0.41, 1.52), p = 0.49). Twelve-month toxicity was affected by subsequent treatments, with no clear differences between arms. Stabilising chemotherapy was associated with poorer PFS (HR: 2.09 (1.14, 3.81), p = 0.017), whereas unfavourable marker decline, in 60 (70%) patients, was not.

**Conclusion:**

Although not powered for PFS, results for CBOP/BEP are promising. Impact on OS was less clear (and will be affected by subsequent therapy). Further study in an international phase III trial is warranted.

**Trial registration:**

ISRCTN 53643604.

## Highlights

• Intensive induction chemotherapy met response targets in poor prognosis germ cell tumours.
• Three-year progression-free survival was 56% for CBOP/BEP and 39% for BEP.
• Impact on survival was less clear and will be affected by subsequent therapy.
• Further study in an international phase III trial is warranted.

## 1 Introduction

BEP (bleomycin, etoposide and cisplatin) chemotherapy has been the standard treatment for metastatic germ cell tumours (GCTs) for many years and is successful for many patients. However, there is a well-characterised poor prognosis group for whom cure rates remain less than 50% [1]. Attempts to improve on this have, to date, been largely unsuccessful [2]. Most recently, the GETUG (Genito-Urinary Group of the French Federation of Cancer Centres) 13 trial suggested that dose intensification based on inadequate marker decline during early treatment may be beneficial [3].

TE23 (ISRCTN 53643604) was a randomised, phase II trial aiming to assess the efficacy and safety of a dose-intense regimen, CBOP/BEP (carboplatin, bleomycin, vincristine, cisplatin/BEP), in these patients. The regimen, originally developed by the Royal Marsden Testicular Tumour Unit based on Wettlaufer *et al.* [4], features early dose-intensity,{super comma?} use of infusional bleomycin (rather than bolus injections) and BEP with reduced bleomycin dose in the second stage.

In the primary analysis, CBOP/BEP met response targets: 74% of patients achieved a complete response or partial response marker negative (90% CI: 61%–85%; primary outcome) [2]. The trial was not powered to compare arms but BEP response rate – used as a benchmark – was as expected at 61% (90% CI: 48%, 73%). Acute toxicity, particularly haematological, was higher with CBOP/BEP.

Here, we report long-term data from the trial, including efficacy outcomes and late toxicity. In addition, the role of early marker decline for predicting long-term outcomes is considered.

## 2 Materials and methods

### 2.1 Patients

Eligible patients were ≥16 years with extracranial GCT and at least one International Germ Cell Cancer Collaborative Group (IGCCCG) poor prognosis feature (mediastinal primary, non-pulmonary visceral metastases, AFP [alpha fetaprotein] > 10,000 ng/ml, HCG [human chorionic gonadotropin] > 50,000iu/l or LDH [lactase dehydrogenase] > 10× upper limit of normal [ULN]). Diagnoses were based on histology; or elevated AFP and/or HCG in a patient with a testicular tumour; or unequivocally elevated markers (AFP>1000 ng/ml or HCG>5000iu/l) in men aged <45 years without a testis tumour but with an otherwise appropriate clinical picture. Applicable regulatory and ethics approvals, and written informed consent, were obtained.

### 2.2 Study design

This was an open-label, parallel, phase II, multicentre, UK trial with randomisation (1:1) to BEP or CBOP/BEP. Eligible patients not deemed fit enough to receive protocol treatment (by the treating investigator) could be stabilised with low-dose chemotherapy (normally 20 mg/m^2^ of cisplatin 20 mg/m or carboplatin AUC3 and etoposide or vincristine for two days) before enrolment. Randomisation at the coordinating trial unit (accessible by telephone to recruiting teams) used minimisation based on preprotocol chemotherapy, primary tumour site, centre and a random element.

### 2.3 Treatment and follow-up assessments

The control arm comprised 4 cycles of Indiana-style 5-day BEP [5] over 12 weeks: 100 mg/m^2^ of etoposide and 20 mg/m^2^ of cisplatin on days 1–5 of each cycle and 12 doses of 30,000iu of bleomycin weekly. The CBOP/BEP arm comprised 6 chemotherapy cycles over 15 weeks. At weeks 1 and 3, 50 mg/m^2^ of cisplatin on days 1 and 2 and 2 mg of vincristine and 15000iu of bleomycin on day 1 were administered. At weeks 2 and 4, 40 mg/m^2^ of cisplatin, 2 mg of vincristine and carboplatin AUC3 all on day 1 and 15000iu of bleomycin by 24-h iv infusion on days 1–5 (total dose 75000iu) were administered. At weeks 5 and 6, 2 mg of vincristine and 15000iu of bleomycin on day 1 were administered. At weeks 7–15, 3 cycles of Indiana-style BEP administered as for the control arm, except with modified bleomycin dose of 15000iu weekly. Prophylactic granulocyte-colony stimulating factor (G-CSF) was mandated (in week five of CBOP/BEP and during each BEP cycle in both arms) from January 2008 following an Independent Data Monitoring Committee recommendation.

Assessments and management during treatment have been reported previously [2]. The protocol specified a 5-year follow-up period after randomisation. Follow-up assessments (clinical examination, chest x-ray and markers) were performed two-monthly in year one, 3-monthly in year two and then six months once to 5 years. Audiometry and lung function were assessed at 12 months and semen analysis at 24 months. Cross-sectional imaging was performed at 2–4 weeks after end of treatment. It was additionally performed to follow residual disease 6-monthly until resolution (<1cm), resected or stable for 1 year; 2 months after surgical resection of tumour masses; and at the investigator's discretion. Surgical resection was advised for all non-resolving masses >1 cm. Management of disease progression was at the clinician's discretion. This analysis was planned when all of the follow-up was complete. For patients who were lost to follow-up, survival status was sought via general practitioners (GPs).

### 2.4 Outcome measures

The primary outcome (reported previously) was favourable response rate: the proportion of patients achieving complete response (disappearance of all disease and normal tumour markers) or partial response (residual mass unresected) with negative markers.

The current analysis focuses on secondary outcomes: progression-free survival (PFS, time from randomisation until disease progression, relapse or death, with treatment failure considered to be an event, and censoring at the most recent assessment for event-free patients); overall survival (OS, time from randomisation until death from any cause, with censoring at the date last known to be alive) and toxicity (Common Terminology Criteria for Adverse Events (CTCAE) v3.0) at 12 months (+/− 2 months). Data on pulmonary and audiometry investigations performed at this time were insufficient.

### 2.5 Statistical analysis

PFS/OS analyses were based on Kaplan-Meier curves and hazard ratios (HRs) derived from Cox regression models with 95% confidence intervals (CIs). Exploratory analyses considered the impact of IGCCCG poor risk features [1], receipt of stabilising chemotherapy and early marker decline, with p < 0.1 (2-sided) regarded as significant. Marker decline was classified based on the GETUG13 algorithm [3,6], considering AFP and HCG values after one BEP cycle (approximately day 21) in the BEP arm or after two CBOP cycles (approximately day 28; with day 14 values used if the later value was not available) in the CBOP/BEP arm (appendix A).

In multivariable models, treatment effect was adjusted for any factors with p < 0.1 in univariate models. Power was limited, particularly for OS, where number of events was small. All randomized patients were included on an intent-to-treat basis with the exception of models incorporating early marker decline, where 3 patients who stopped treatment before the relevant marker assessment were excluded. Subgroup analyses considered the impact of early marker decline in the BEP arm and in those who did not receive stabilising chemotherapy.

Late toxicity data are tabulated by treatment arm for surviving patients (regardless of subsequent treatments) with no formal comparative tests.

Analyses were performed using Stata (StataCorp), version 14.

## 3 Results

**Fig. 1:**
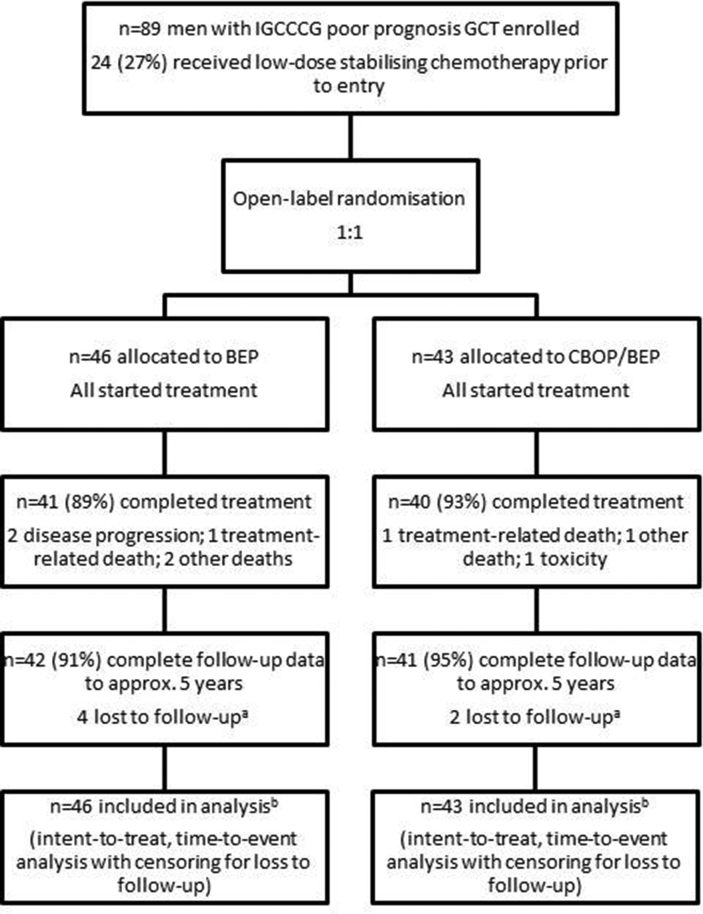
Flow of participants through the trial. ^a^ 2 of these patients had already had a progression event at the time of loss to follow-up (1 BEP and 1 CBOP/BEP, with 38 and 9 month postprogression follow-up, respectively) and so contribute complete data to the PFS analysis; in addition, survival information at ≥5 years was obtained from GPs for the 2 patients who had already progressed and a further 1 BEP patient. ^b^ 3 patients (2 BEP, 1 CBOP/BEP) are excluded from models incorporating marker decline because they only received one cycle of treatment in the trial and, therefore, did not have the relevant marker assessment for calculation of time to normalisation. In addition, analyses of 12-month toxicity are restricted to surviving patients (33 BEP, 32 CBOP/BEP) with follow-up assessment data between 10 and 14 months after randomisation.

Between June 2005 and December 2009, 89 patients were enrolled from 16 UK centres (recruiting between 1 and 15 patients each, median 5); 43 of them were randomised to CBOP/BEP (Fig. 1). The mean age was 30 years (range 16–68), and 18 patients (20%) had mediastinal primary tumours. Fifty-three patients (60%) had diagnosis confirmed histologically and 36 (40%) based on markers/clinical picture. Sixty-four patients (72%) had not undergone orchidectomy at the time of trial registration. Twenty-four patients (27%) had low-dose chemotherapy for stabilisation. IGCCCG poor prognosis features and receipt of stabilising chemotherapy were similar between arms (Table 1). Details of treatment and acute toxicity have been reported previously: completion rates were similarly high in the two arms (41/46, 89% for BEP; 40/43, 93% CBOP/BEP); rates of CTCAE grade ≥3 symptoms were higher with CBOP/BEP (95% vs 63% with BEP), largely because of haematological toxicity.

**Table 1:** Baseline characteristics.

Baseline data		BEP (n = 46)		CBOP/BEP (n = 43)		Overall (n = 89)	
		No.	(%)	No.	(%)	No.	(%)
Age (years)	Mean (range)	31 (16–68)		28 (16–60)		30 (16–68)	
Site of primary tumour	Testis	34	(72)	32	(74)	66	(74)
	Mediastinum	9	(20)	9	(21)	18	(20)
	Retroperitoneum	2	(4)	2	(5)	4	(4)
	Unclear^a^	1	(4)	0	(0)	1	(1)
IGCCCG poor risk factors	Raised markers^b^ only	14	(30)	10	(23)	24	(27)
	Mediastinal primary only	7	(15)	4	(9)	11	(12)
	Non-pulmonary visceral metastases (NPVM) only	9	(20)	10	(23)	19	(21)
	Raised markers and mediastinal primary	2	(4)	3	(7)	5	(6)
	Raised markers and NPVM	14	(30)	14	(33)	28	(31)
	Mediastinal primary and NPVM	0	(0)	2	(5)	2	(2)
Orchidectomy before registration	No	34	(74)	30	(70)	64	(72)
	Yes	12	(26)	13	(30)	25	(28)
Receipt of stabilising chemotherapy before protocol treatment	No	33	(72)	32	(74)	65	(73)
	Yes	13	(28)	11	(26)	24	(27)

CBOP/BEP, carboplatin, bleomycin, vincristine, cisplatin/BEP; BEP, bleomycin, etoposide and cisplatin; LDH, lactase dehydrogenase; AFP, alpha fetaprotein; HCG, human chorionic gonadotropin.

a Difficult to determine between the testis and retroperitoneum.

b AFP>10,000iu/l, HCG>50,000iu/l or LDH>10 × upper limit of normal.

Median follow-up was 63 months – similar between arms. All surviving patients had a minimum 58-month follow-up, with the exception of 6 who were lost to follow-up (4 BEP, 2 CBOP/BEP). Of these, 2 had had progression events (1 BEP treatment failure, with 38-month post-failure follow-up; 1 progression following CBOP/BEP, with 9 months follow-up thereafter). Survival status at ≥5 years was obtained through GPs for these 2 patients, and for a furtherBEP patient who was lost to follow-up.

### 3.1 Marker decline

Time to normalisation could not be calculated for 3 patients (2 BEP, 1 CBOP/BEP) who only received one cycle of treatment in the trial and so did not have the relevant marker assessment. Sixty of eighty-six (70%) of patients had unfavourable marker decline, and this was similar in the two arms (BEP: 31/44, 70.5%, 90% CI: 57.2%–81.6%; CBOP/BEP: 29/42, 69.0%, 90% CI 55:.4%–80.6%). As might be expected, high baseline AFP or HCG was correlated with unfavourable marker decline. The reverse trend was seen for LDH – a small number of patients had high (>10 ULN) LDH values, and these patients were more likely to have favourable marker decline. There were no other associations between IGCCCG factors or receipt of stabilising chemotherapy and marker decline (appendix B).

### 3.2 PFS and OS

**Fig. 2:**
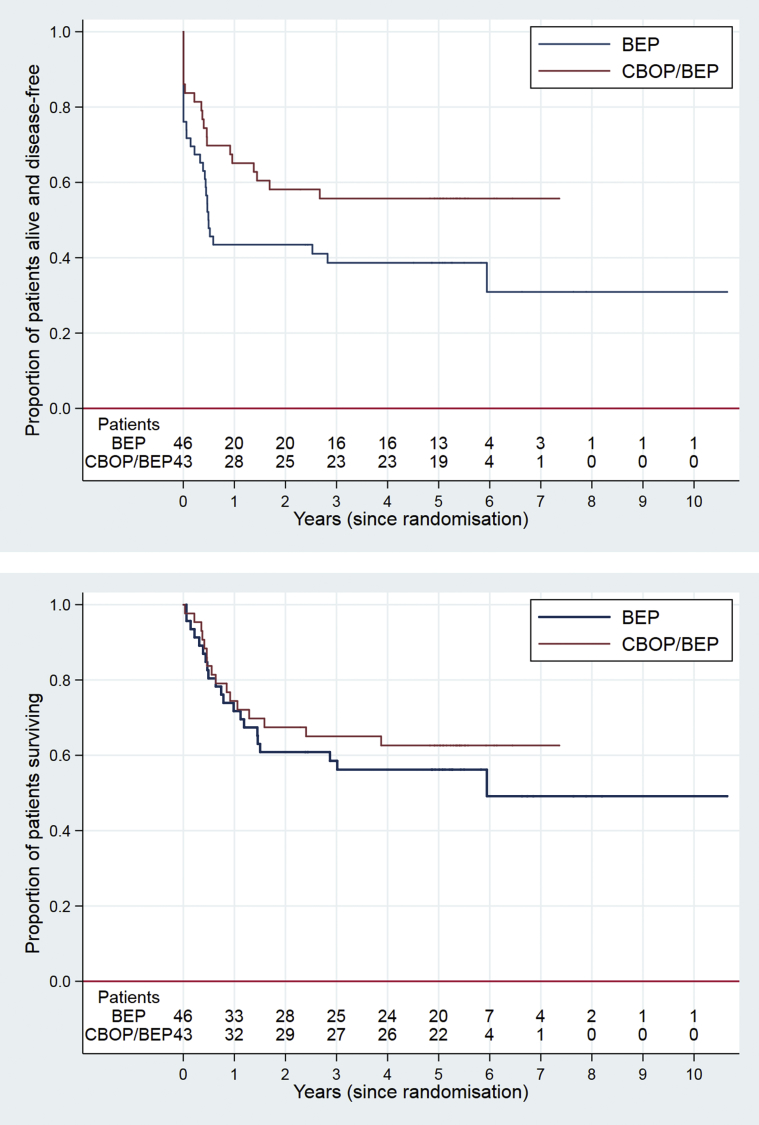
(a) Progression-free survival according to treatment allocation; (b) Overall survival according to treatment allocation.

There were 48 PFS events (29 BEP, 19 CBOP/BEP), and median PFS was 30 months (6 months in the BEP arm, not yet reached in the CBOP/BEP arm). Estimated 3-year PFS was 38.7% (95% CI: 24.7%–52.4%) in the BEP arm and 55.7% (39.7%–69.0%) for the CBOP/BEP arm; hazard ratio (HR) = 0.59 (0.33–1.06), p = 0.079 (Fig. 2a). Rates remained the same at 5 years.

There were 37 deaths (21 BEP, 16 CBOP/BEP). Median survival time was just less than 6 years in the BEP arm and had not yet been reached for the CBOP/BEP arm. Estimated 3-year OS was 58.5% (43.0%–71.2%) in the BEP arm and 65.0% (48.8%–77.2%) for the CBOP/BEP arm; HR = 0.79 (95% CI: 0.41 to 1.52), p = 0.49 (Fig. 2b).

### 3.3 Prognostic models

Estimated 3-year PFS was higher in the group with unfavourable marker decline compared with those with favourable decline, though this trend was non-significant (51.7% vs 41.0%, HR = 0.82, p = 0.54). Results were similar in the subgroup who did not receive stabilising chemotherapy and in the subgroup of BEP patients.

In univariate models, receipt of stabilising chemotherapy was associated with poorer PFS (Table 2). No significant associations were observed for IGCCCG poor risk factors, although there were non-significant negative trends associated with mediastinal primary site and multiple IGCCCG poor prognosis features. Treatment effect remained similar after adjustment for stabilising chemotherapy (Table 2).

**Table 2:** Univariate and multivariable models for predicting progression-free survival.

Factor		No.	Univariate HR (95% CI) – Cox model	P-value	Multivariable HR (95% CI)	P-value
Trial arm	BEP	46	Ref	0.079	Ref	0.074
	CBOP/BEP	43	0.59 (0.33, 1.06)		0.59 (0.33, 1.05)	
AFP	≤10000 ng/ml	65	Ref	0.17	–	–
	>10000ng/ml	24	0.62 (0.31, 1.24)			
HCG	≤50000 iu/l	58	Ref	0.40	–	–
	>50000 iu/l	31	1.28 (0.72, 2.31)			
LDH	≤10xULN	80	Ref	0.63	–	–
	>10xULN	9	1.24 (0.52, 2.91)			
IGCCCG poor prognosis markers	No	32	Ref	0.48	–	–
	Yes^a^	57	0.81 (0.46, 1.45)			
Mediastinal primary site	No	71	Ref	0.39	–	–
	Yes	18	1.34 (0.68, 2.64)			
Non-pulmonary visceral mets	No	40	Ref	0.42	–	–
	Yes	49	1.27 (0.71, 2.26)			
Multiple IGCCCG poor prognosis factors	No	52	Ref	0.20	–	–
	Yes^b^	37	1.45 (0.82, 2.55)			
Preprotocol chemotherapy	No	65	Ref	0.018	Ref	0.017
	Yes	24	2.07 (1.13, 3.77)		2.09 (1.14, 3.81)	
Early marker decline	Favourable	26	Ref	0.54	–	–
	Unfavourable	60	0.82 (0.44, 1.53)			

LDH, lactase dehydrogenase; AFP, alpha fetaprotein; HCG, human chorionic gonadotropin; CI, confidence interval; HR, hazard ratio; CBOP/BEP, carboplatin, bleomycin, vincristine, cisplatin/BEP; BEP, bleomycin, etoposide and cisplatin; ULN, upper limit of normal.

a One or more of: AFP>10000 ng/ml, HCG>50,000iu/l or LDH>10xULN.

b Two or more of: mediastinal primary; non-pulmonary visceral mets; AFP>10000 ng/ml; HCG>50,000iu/l; LDH>10xULN.

Estimated 3-year OS was higher in the group with unfavourable marker decline compared with those with favourable decline, although this was non-significant because of the low power of the model (66.7% vs 57.7%, HR = 0.59, p = 0.14). Results were similar in the two subgroup analyses.

Results for univariate and multivariable models for OS were similar to those for PFS – the only significant associations were for receipt of stabilising chemotherapy and mediastinal primary tumours (appendix C). Treatment effect remained similar after adjustment for these factors.

### 3.4 Late toxicity

At 12 months, there were 65 surviving patients (33 BEP, 32 CBOP/BEP), of which 11 (7 BEP, 4 CBOP/BEP) had active disease. Burden of toxicity was similarly low in both arms (Table 3). CTCAE grade ≥3 symptoms were reported in five patients in the BEP arm, all of whom had recently undergone further subsequent chemotherapy, and so symptoms may have been related to that treatment (or to active disease).

**Table 3:** Toxicity reported at 12 months (+/− 2 months) after randomisation.

		BEP (n = 33)		CBOP/BEP (n = 32)	
	CTCAE (v3.0) grade	No.	(%)	No.	(%)
Status	Alive no disease	7	(21)	13	(41)
	Alive inactive disease	19	(58)	15	(47)
	Alive active disease	7	(21)	4	(13)
Dermatological symptoms	0	29	(88)	29	(91)
	1	0	(0)	2	(6)
	2	1	(3)	0	(0)
	Missing	3	(9)	1	(3)
Haematological toxicity	0	28	(85)	29	(91)
	1	1	(3)	1	(3)
	2	0	(0)	1	(3)
	3	0	(0)	0	(0)
	4	1	(3)	0	(0)
	Specific (grade 4)	Hb 5.9			
	Missing	3	(10)	0	(0)
Pulmonary symptoms	0	26	(79)	30	(94)
	1	1	(3)	1	(3)
	2	2	(6)	0	(0)
	3	1	(3)	0	(0)
	Specific (grade 3)	Shortness of breath			
	Missing	3	(9)	1	(3)
Fatigue	0	23	(70)	25	(78)
	1	6	(18)	4	(13)
	2	0	(0)	2	(6)
	3	1	(3)	0	(0)
	Missing	3	(9)	1	(3)
Cardiovascular symptoms	0	30	(91)	31	(97)
	Missing	3	(9)	1	(3)
Vascular symptoms	0	30	(91)	30	(94)
	1	0	(0)	1	(3)
	Missing	3	(9)	1	(3)
Renal impairment	0	29	(88)	31	(97)
	1	0	(0)	0	(0)
	2	1	(3)	0	(0)
	Missing	3	(9)	1	(3)
Anorexia/Weight loss	0	27	(82)	30	(94)
	1	1	(3)	0	(0)
	2	1	(3)	1	(3)
	Missing	4	(12)	1	(3)
Sensory neuropathy	0	23	(70)	19	(59)
	1	3	(9)	9	(28)
	2	3	(9)	3	(9)
	3	0	(0)	0	(0)
	4	1	(3)	0	(0)
	Missing	3	(9)	1	(3)
Auditory changes/tinnitus symptoms	0	27	(82)	24	(75)
	1	1	(3)	5	(16)
	2	1	(3)	2	(6)
	3	1	(3)	0	(0)
	Missing	3	(9)	1	(3)
Other symptoms	0	25	(76)	30	(94)
	1	3	(9)	1	(3)
	2	1	(3)	0	(0)
	3	1	(3)	0	(0)
	Specific (grade 3)	Lumbar pain			
	Missing	3	(9)	1	(3)

CBOP/BEP, carboplatin, bleomycin, vincristine, cisplatin/BEP; BEP, bleomycin, etoposide and cisplatin.

### 3.5 Treatment at relapse

Thirty-five patients relapsed or their disease progressed during the trial (22 BEP, 13 CBOP/BEP), including all patients with a treatment failure. Thirty patients (20 BEP, 10 CBOP/BEP) received treatment for relapse. Of the remaining five, three died around the time of diagnosis of relapse/progression; one was felt not to be fit enough for salvage treatment and died two months later; and the remaining patient did not require further treatment and remained alive at the end of the follow-up. Of those treated, all but 5 received combination chemotherapy (sometimes alongside radiotherapy or surgery), most commonly paclitaxel, ifosfamide and cisplatin (TIP) (13 patients), but a range of other regimens were used. Only 1 patient (BEP arm) had a complete response and 9 (7 BEP, 2 CBOP/BEP) had a partial response with negative markers.

## 4 Discussion

Long-term data from this randomised, phase II trial support the conclusion of the primary analysis, which demonstrated that CBOP/BEP met response targets and was feasible to deliver across UK centres. Although the trial was not powered to compare efficacy outcomes, a trend suggesting a PFS benefit was observed (3-year PFS 56% vs 39% on BEP, HR = 0.59, p = 0.079). Survival rates in the two arms were more similar, although slightly higher with CBOP/BEP. This is to be expected given that survival rates will be influenced by subsequent treatment. Twice as many patients received salvage treatment after BEP (20/46, 44%, vs 10/43, 23%, after CBOP/BEP). This phenomenon of fewer patients in the dose-intense arm was also seen in the GETUG13 trial [3]. Alhough numbers are small and differences are not statistically significant, a higher proportion of patients achieved a favourable response to salvage chemotherapy (BEP: 8/20, 40%; CBOP/BEP: 2/10, 20%) which will contribute to a smaller difference between arms in OS when compared with PFS. However, the burden of salvage therapy, both physical and psychological, may add to the justification for more intensive upfront approaches. In the primary analysis of TE23, CBOP/BEP was associated with an increase in acute haematological toxicity, but current data provide reassurance that there are no late effects.

Our previous systematic review highlighted that – despite a number of phase II and III randomised trials of first-line treatment for intermediate and poor prognosis of GCTs conducted internationally in recent years – no single approach has emerged as clearly superior to BEP [2]. A notable exception is the GETUG13 trial, which demonstrated a PFS benefit in a subgroup of poor risk patients with inadequate early marker decline who were switched to a dose-dense regimen [3]. Three-year PFS was similar to that observed with CBOP/BEP (59% vs 48% on BEP, p = 0.05) and – as for CBOP/BEP – impact on survival was less clear. Results for high-dose VIP (cisplatin, etoposide and ifosfamide) in EORTC30974, although non-significant, were also similar (2-year failure-free survival 58% vs 45%) [7] – but other high-dose chemotherapy regimens have shown less promise [[8], [9], [10]].No improvement in response rates was seen with TIP (paclitaxel, ifosfamide and cisplatin) when compared with BEP in a recent randomised phase II trial (76% vs 73%; 1-year PFS 72% both arms) [11].

CBOP/BEP takes a different approach to other treatments by delivering intensification upfront. An advantage of this approach is that maximal therapy is delivered when the cancer is at its most bulky, enabling a more rapid control of symptoms and potentially also reducing the development of resistance. Additional benefits, from a patient perspective, are that the regimen may be modified to fit the individual's clinical situation and the intensive phase is given when patients are often in hospital with disease-related morbidity. One difficulty is that it does not fit well with the approach of picking patients for intensive treatment based on marker decline. A further dose-intense approach, demonstrating potential in early phase, single arm studies, is accelerated BEP [12,13], and results from the phase III ANZUP 1302 trial (NCT02582697) are eagerly awaited [14]. For all of these strategies, possible risks of increased toxicity must be carefully weighed against potential benefits, and more long-term data are needed to provide holistic assessments.

A factor in the lack of success of some previous trials is that recruitment potential does not match the need. It is also important to be realistic about what benefits are achievable, particularly in terms of survival, which will be influenced by subsequent treatment and events. Some previous studies have been underpowered to detect realistic effects or have failed to recruit to target. Accrual to TE23 – across 16 UK centres – took twice as long as originally anticipated (4 years rather than 2 years), hence not meeting the criteria for immediate progression to a phase III study. Clearly, major international collaborations will be required to recruit to well-powered phase III trials in a timely manner. In addition, future trials in this patient group should look to novel methodology for evaluating treatments for rarer cancers, particularly given that there are a number of treatment strategies which might benefit from simultaneous evaluation [15].

Another factor in evaluating treatment strategies is patient selection. For many years, IGCCCG criteria have provided a simple system – based on a large, international data set – for identifying patients for whom standard BEP treatment is less likely to be successful [1], and trials have focused on these groups. However, this classification is now based on data from several decades ago. In the current analysis, as in other studies, there is the suggestion of heterogeneity within the poor risk group and a need to identify a ‘poor poor risk’ group. As part of an international effort to update the classification system, Gillessen *et al.* [16] have recently proposed the use of age and lung metastases as additional factors based on a large, international, pooled data set. If validated in an independent data set (work which is currently underway), this will facilitate more targeted treatment approaches.

The TE23 cohort included a high proportion (40%) of men diagnosed on the basis of markers/clinical picture, rather than histology, and a substantial number of patients (27%) were deemed to require stabilising chemotherapy before full-dose treatment. As such, they may represent a particularly poor risk group, and this is reflected in poorer outcomes for BEP (5-year PFS and OS: 39% and 56%, respectively) relative to contemporary series, including the pooled data set mentioned previously [[16], [17], [18]]. Outcomes with CBOP/BEP (5-year PFS and OS 56% and 63% respectively) were more similar to these series, with improved PFS and OS relative to the original IGCCCG study (published in 1997). As in the other contemporary studies, even for the BEP arm of TE23, an improvement in OS was seen relative to this earlier study, suggesting an impact of better salvage treatment.

In TE23, use of stabilising chemotherapy was the factor most clearly associated with poor outcomes. The most likely explanation for this is that the clinician's assessment of disease at the outset – which may not be fully captured with current risk factors – may provide the best indicator of the likely success of treatment. However, we can not exclude a detrimental impact of the use of initial low-dose therapy/delay in commencing full-dose therapy.

Targeting more intensive treatment at those patients most likely to benefit is desirable. One strategy is to select patients based on marker decline after a single BEP cycle. Within the IGCCCG poor risk group, inadequate marker decline has been shown to be independently associated with worse PFS and OS [6] and, in a subsequent trial (GETUG13), this group benefitted from switching to a dose-dense regimen [3]. In TE23, however, inadequate marker decline was not associated with outcomes. It is possible that early marker decline could be less relevant in patients treated with CBOP/BEP – however, the BEP subgroup analysis produced similar results. This lack of association could be due to the modest size of this study or may suggest that the predictive value of early marker decline is less valuable in a patient population with very poor prognostic disease. Further larger studies exploring this heterogeneity would be helpful to establish in which patients’ marker decline is most/least relevant.

A limitation of this study is the small sample size, which is insufficient for complex multivariable modelling. The lack of a clear effect of early marker decline may reflect this – although it is notable that no trend was present. A further limitation is that outcome data beyond 5 years were not collected, although it is notable that few events (either PFS or OS) occurred after 3 years. A strength of this study is the randomised nature, providing a contemporary group of patients treated with BEP, and largely complete follow-up data up to 5 years.

## 5 Conclusion

CBOP/BEP has shown promise for treating men with poor prognosis GCT in a randomised, phase II trial – response targets were met, and a PFS benefit is suggested. Impact on survival is less clear and will be influenced by subsequent treatment. The regimen warrants study in a phase III trial.

## Role of the funding source

Cancer Research UK had no role in the study design; the collection, analysis and interpretation of data; the writing of the report; nor the decision to submit for publication.

## Conflict of interest statement

R.A.H. reports activities outside the submitted work, including receiving non-financial support from Janssen, grants and personal fees from MSD, personal fees from Bristol Myers Squibb, grants from CRUK, advisory board fees and travel expenses from Nekta and personal fees and non-financial support from Roche. F.H.C., J.D.W., J.S., I.H. and S.P.S. have nothing to disclose.
