# Supplementary material for: Long-term outcomes with intensive induction chemotherapy (carboplatin, bleomycin, vincristine and cisplatin/bleomycin, etoposide and cisplatin) and standard bleomycin, etoposide and cisplatin in poor prognosis germ cell tumours: A randomised phase II trial (ISRCTN53643604)
Source: Eur J Cancer. 2020 Mar;127:139–49. doi: 10.1016/j.ejca.2019.12.028 (PMC7045084; doi:10.1016/j.ejca.2019.12.028)
Supplement: Multimedia component 1 [file mmc1.docx]

**APPENDICES**

**Appendix A: Definitions for classifying marker decline during early treatment**

Marker decline was classified using an algorithm adapted from that used in the GETUG13 trial[3, 6], based on marker values (AFP and HCG) during early treatment cycles. For each of the markers (M), the theoretical time to normalisation (TN_M_) is calculated using the formula below and is used to classify patients into one of the four groups (A-D). Patients with a favourable decline are defined as those who fall into categories A, B or C for both AFP and HCG. Those with an unfavourable marker decline are any patients falling into category D for either AFP or HCG (or both).

**Notation**

TN_M_ = Time to normalisation of marker M

M_0_ = Pre-treatment (day 0) marker value from just prior to cycle 1

M_1_ = Marker value during early treatment (as defined below)

W = Weeks between marker measurements M_0_ and M_1_

M_N_ = centre-specific upper limit of normal for marker M

T_M_ = Time to normalisation cut-off point (weeks); T_AFP_=9, T_HCG_=6

**Marker values during treatment (M_1_)**

For BEP patients, the marker value obtained prior to the 2^nd^ BEP cycle (approximately day 21) is used; for CBOP/BEP the marker value obtained prior to the BO cycle (approximately day 28) will be used. W is, therefore, 3 and 4 respectively for the two treatment groups. Note that exact dates of marker evaluation were not collected on case report forms, so all timings are approximate. In the event that day 28 data is not available for a CBOP/BEP patient, the marker value from prior to the 2^nd^ CBOP cycle (approximately day 14) is used and, in that case, W is 2.

**Formula**

TN_M_ = W x (log_10_(M_0_) – log_10_(M_N_))/(log_10_(M_0_) – log_10_(M_1_))

**Marker decline groups**

A_M_: Normal values for M_0_ and M_1_

B_M_: Raised M_0_, normal M_1_ value

C_M_: Raised M_0_ and TN_M_ < T_M_

D_M_: Raised M_0_ and TN_M_ ≥ T_M_; or raised M_0_ and M_0_<M_1_ (increasing)

**Details of derivation**

Time to normalisation could not be calculated for 3 patients (2 BEP, 1 CBOP/BEP) who only received one cycle of treatment in the trial and so did not have the relevant marker assessment. Additionally, for 6 patients, marker decline group could not be assigned for one of the markers. In one case (BEP patient) this was due to missing the relevant AFP value. The other 5 patients had a normal pre-treatment AFP which had subsequently increased to above the upper limit of normal during early treatment, but with calculated time to normalisation below the threshold value. This scenario does not fit any of the categories defined above. Nevertheless, in all 6 cases, the marker decline category could be determined by the decline in HCG (which was unfavourable).

**Appendix B: Early marker decline according to baseline risk factors and treatment allocation**

|  | | | | **Favourable marker decline (n=26)** | | **Unfavourable marker decline (n=60)** | |
| --- | --- | --- | --- | --- | --- | --- | --- |
|  | |  | | **No.** | **(%)** | **No.** | **(%)** |
| **AFP** | ≤10000 ng/ml | | | 23 | (36) | 41 | (64) |
|  | >10000 ng/ml | | | 3 | (14) | 19 | (86) |
|  |  | | |  |  |  |  |
| **HCG** | ≤50000 iu/l | | | 22 | (39) | 34 | (61) |
|  | >50000 iu/l | | | 4 | (13) | 26 | (87) |
|  |  | | |  |  |  |  |
| **LDH** | ≤10xULN | | | 21 | (27) | 57 | (73) |
|  | >10xULN | | | 5 | (63) | 3 | (38) |
|  |  | | |  |  |  |  |
| **Mediastinal primary** | No | | | 21 | (30) | 48 | (70) |
|  | Yes | | | 5 | (29) | 12 | (71) |
|  |  | | |  |  |  |  |
| **Non-pulmonary visceral mets** | | | No | 10 | (26) | 28 | (74) |
|  | | | Yes | 16 | (33) | 32 | (67) |
|  | | |  |  |  |  |  |
| **Receipt of pre-protocol chemo** | | | No | 18 | (28) | 46 | (72) |
|  | | | Yes | 8 | (36) | 14 | (64) |
|  | | |  |  |  |  |  |
| **Trial arm** | | | BEP | 13 | (30) | 31 | (70) |
| CBOP/BEP | | | | 13 | (31) | 29 | (69) |
|  | | | |  |  |  |  |

**Appendix C: Univariate and multivariable models for predicting overall survival**

| **Factor** |  | **No.** | **Univariate HR (95% CI) – Cox model** | **P-value** | **Multivariate HR (95% CI)** | **P-value** |
| --- | --- | --- | --- | --- | --- | --- |
| Trial arm | BEP | 46 | Ref | 0.49 | Ref | 0.38 |
|  | CBOP/BEP | 43 | 0.79 (0.41, 1.52) |  | 0.75 (0.39, 1.44) |  |
|  |  |  |  |  |  |  |
| AFP | ≤10000 ng/ml | 65 | Ref | 0.69 | - | - |
|  | >10000ng/ml | 24 | 0.86 (0.41, 1.82) |  |  |  |
|  |  |  |  |  |  |  |
| HCG | ≤50000 iu/l | 58 | Ref | 0.95 | - | - |
|  | >50000iu/l | 31 | 0.98 (0.50, 1.92) |  |  |  |
|  |  |  |  |  |  |  |
| LDH | ≤10xULN | 80 | Ref | 0.40 | - | - |
|  | >10xULN | 9 | 1.50 (0.58, 3.86) |  |  |  |
|  |  |  |  |  |  |  |
| IGCCCG poor prognosis markers | No | 32 | Ref | 0.42 | - | - |
|  | Yes^a^ | 57 | 0.76 (0.40, 1.47) |  |  |  |
|  |  |  |  |  |  |  |
| Mediastinal primary site | No | 71 | Ref | 0.098 | Ref | 0.045 |
|  | Yes | 18 | 1.85 (0.89, 3.83) |  | 2.13 (1.02, 4.46) |  |
|  |  |  |  |  |  |  |
| Non-pulmonary visceral mets | No | 40 | Ref | 0.64 | - | - |
|  | Yes | 49 | 1.17 (0.61, 2.26) |  |  |  |
|  |  |  |  |  |  |  |
| Multiple IGCCCG poor prognosis factors | No | 52 | Ref | 0.14 | - | - |
|  | Yes^b^ | 37 | 1.62 (0.85, 3.08) |  |  |  |
|  |  |  |  |  |  |  |
| Pre-protocol chemotherapy | No | 65 | Ref | 0.001 | Ref | <0.001 |
|  | Yes | 24 | 3.19 (1.65, 6.17) |  | 3.40 (1.74, 6.63) |  |
|  |  |  |  |  |  |  |
| Early marker decline | Favourable | 26 | Ref | 0.14 | - | - |
|  | Unfavourable | 60 | 0.59 (0.30, 1.18) |  |  |  |
|  |  |  |  |  |  |  |

^a^ One or more of: AFP>10000ng/ml, HCG>50,000iu/l or LDH>10xULN.

^b^ Two or more of: mediastinal primary; non-pulmonary visceral mets; AFP>10000ng/ml; HCG>50,000iu/l; LDH>10xULN.
